# Sex Differences in Skeletal Muscle Pathology in Patients With Heart Failure and Reduced Ejection Fraction

**DOI:** 10.1161/CIRCHEARTFAILURE.123.011471

**Published:** 2024-10-09

**Authors:** Nathanael Wood, Annabel Critchlow, Chew W. Cheng, Sam Straw, Paul W. Hendrickse, Marcelo G. Pereira, Stephen B. Wheatcroft, Stuart Egginton, Klaus K. Witte, Lee D. Roberts, T. Scott Bowen

**Affiliations:** Faculty of Biological Sciences, School of Biomedical Sciences (N.W., A.C., M.G.P., S.E., T.S.B.), University of Leeds, United Kingdom.; Leeds Institute of Cardiovascular and Metabolic Medicine (C.W.C., S.S., S.B.W., K.K.W., L.D.R.), University of Leeds, United Kingdom.; Medical Faculty, Lancaster University, United Kingdom (P.W.H.).; Clinic for Cardiology, Angiology and Internal Intensive Care Medicine, RWTH Aachen University, Germany (K.K.W.).

**Keywords:** atrophy, capillaries, exercise, muscles, sarcopenia, women

## Abstract

**BACKGROUND:**

Women with heart failure and reduced ejection fraction (HFrEF) have greater symptoms and a lower quality of life compared with men; however, the role of noncardiac mechanisms remains poorly resolved. We hypothesized that differences in skeletal muscle pathology between men and women with HFrEF may explain clinical heterogeneity.

**METHODS:**

Muscle biopsies from both men (n=22) and women (n=16) with moderate HFrEF (New York Heart Association classes I–III) and age- and sex-matched controls (n=18 and n=16, respectively) underwent transcriptomics (RNA-sequencing), myofiber structural imaging (histology), and molecular signaling analysis (gene/protein expression), with serum inflammatory profiles analyzed (enzyme-linked immunosorbent assay). Two-way ANOVA was conducted (interaction sex and condition).

**RESULTS:**

RNA-sequencing identified 5629 differentially expressed genes between men and women with HFrEF, with upregulated terms for catabolism and downregulated terms for mitochondria in men. mRNA expression confirmed an effect of sex (*P*<0.05) on proatrophic genes related to ubiquitin proteasome, autophagy, and myostatin systems (higher in all men versus all women), whereas proanabolic *IGF1* expression was higher (*P*<0.05) in women with HFrEF only. Structurally, women compared with men with HFrEF showed a pro-oxidative phenotype, with smaller but higher numbers of type I fibers, alongside higher muscle capillarity (*P*_interaction_<0.05) and higher type I fiber areal density (*P*_interaction_<0.05). Differences in gene/protein expression of regulators of muscle phenotype were detected between sexes, including *HIF1α*, *ESR1*, *VEGF* (vascular endothelial growth factor), and PGC1α expression (*P*<0.05), and for upstream circulating factors, including VEGF, IL (interleukin)-6, and IL-8 (*P*<0.05).

**CONCLUSIONS:**

Sex differences in muscle pathology in HFrEF exist, with men showing greater abnormalities compared with women related to the transcriptome, fiber phenotype, capillarity, and circulating factors. These preliminary data question whether muscle pathology is a primary mechanism contributing to greater symptoms in women with HFrEF and highlight the need for further investigation.

**WHAT IS NEW?**

- Many people with heart failure (HF) develop skeletal muscle pathology (sarcopenia), which increases symptom burden. The influence of sex differences on this unmet clinical need remained poorly addressed.
- By directly sampling muscle tissue, this study found differences in the severity of muscle pathology developed between men and women with HF and reduced ejection fraction.
- Compared with women, men with HF and reduced ejection fraction showed greater muscle pathology that ranged from changes at the molecular gene level to alterations in the structural composition of the muscle.

**WHAT ARE THE CLINICAL IMPLICATIONS?**

- Clinicians should consider that the cause of symptoms in men compared with women with HF and reduced ejection fraction may be explained by more peripheral muscle abnormalities.
- Compared with women, men with HF and reduced ejection fraction may benefit more from therapeutic interventions that specifically target poor muscle health.
- Identifying sex-specific skeletal muscle therapies in the future may provide more effective treatments for reducing symptoms in men and women with HF.

Heart failure (HF) remains an incurable disease associated with high mortality rates and low quality of life.^1^ The overwhelming majority of patients suffer persistent symptoms of breathlessness and fatigue despite contemporary therapies,^2^ leading to exercise intolerance and poor quality of life. An important consideration for improving our understanding and treatment of HF and reduced ejection fraction (HFrEF) is sex differences.^3^ However, underrepresentation of women in HFrEF studies remains a problem,^4,5^ despite women accounting for ≈30% to 40% of this disease population.^6,7^ Importantly, women have different clinical pathophysiology to men with HFrEF, showing lower hospitalization and mortality rates but higher physical disability, worse symptoms, and a lower health-related quality of life.^8–10^ Despite men and women with HFrEF presenting with similar degrees of cardiac dysfunction,^9^ women do not always respond favorably to cardiocentric treatments of proven benefit in men^11^ and participate in less exercise rehabilitation.^9^ Collectively, current evidence indicates that sex differences related to noncardiac mechanisms in HFrEF could play an important role in disease heterogeneity.

In this regard, skeletal muscle abnormalities in HFrEF have emerged as an important cause and treatment target in disease progression.^12^ At present, however, few studies have comprehensively addressed the impact of sex on muscle pathology in HFrEF and those performed showed conflicting findings with various experimental shortcomings.^13–15^ Therefore, the majority of our knowledge regarding skeletal muscle pathology in HFrEF is derived from male patients (eg, characterized by fiber atrophy, an increased proportion of more fatigable type II fibers, low muscle capillarity, impaired mitochondrial function, and molecular alterations related to shifts in procatabolic and proinflammatory signaling),^16^ which may not translate to women. This was recently supported by a study measuring exercise hemodynamics that concluded women likely develop increased muscle abnormalities than men in HF with preserved ejection fraction.^17^

Based on evidence that skeletal muscle pathology directly exacerbates exercise intolerance^18^ and is a strong independent predictor of symptoms^19^ and mortality in patients with HFrEF,^20^ the current study aimed to investigate if muscle pathology in HFrEF is influenced by sex. To address this, we analyzed muscle biopsies samples from both men and women with HFrEF in parallel to age- and sex-matched controls and then performed high-throughput transcriptomics, myofiber and capillarity structural imaging, muscle phenotype molecular signaling analysis, and upstream systemic inflammatory profiling. As women compared with men with HFrEF are reported to have greater physical limitations, more symptoms, and a lower quality of life,^9^ we hypothesized that higher muscle pathology could be a contributory factor.

## METHODS

### Data Availability

The data that support the findings of this study are available from the corresponding author upon reasonable request.

#### Patients

Male (n=22) and female (n=16) patients with established HFrEF, who had persistent symptoms and a left ventricular ejection fraction <40% (as confirmed by echocardiography despite having received at least 3 months of guideline-directed medical therapy, in line with international guidelines)^21^ undergoing routine cardiac implantable electronic device implantation at Leeds Teaching Hospitals, were approached to take part in this study. Patients with no evidence of HF (left ventricular ejection fraction >40%) but requiring cardiac implantable electronic device surgery served as controls (n=18 males; n=16 females) due to sinus node dysfunction, atrial fibrillation, or atrioventricular block. All participants provided written informed consent, and all procedures were conducted in accordance with the Declaration of Helsinki after receiving local institute ethical approval (11/YH/0291). During cardiac implantable electronic device implantation, skeletal muscle biopsies from the pectoralis major were collected from each patient (between 2015 and 2020), frozen in liquid nitrogen, and stored at −80 °C. Subsequent muscle analyses were then performed including myofiber structural imaging, transcriptomics, gene and protein expression, and serum profiling. Full details of these approaches are provided within the Supplemental Material.

### Statistical Analysis

Data were analyzed in Prism (GraphPad Prism 9, v9.4.1). The Maurice test of sphericity was conducted to assess normality. Two-way ANOVAs were conducted to assess differences between condition and sex. Tukey multiple comparison tests were conducted as part of the 2-way ANOVA to assess differences between all groups. The *t* tests were conducted for within-group comparisons. The χ^2^ tests were used to compare categorical clinical variables. Comparisons between controls of one sex to patients with HFrEF of the other sex (ie, male controls versus female HFrEF or female controls versus male HFrEF) were not included as these were not directly relevant to the study aims and hypotheses. Statistical significance was accepted at *P*<0.05, and data are presented as min-max box plots with median, quartile ranges, and the mean displayed unless stated otherwise.

## RESULTS

### Patient Characteristics

Patient characteristics are shown in Table 1. There were no differences (*P*>0.05) between age, body mass index, and glycated hemoglobin between the 4 groups, whereas men and women with HFrEF had similar degrees of cardiac impairment (Table 1).

**Table 1.**
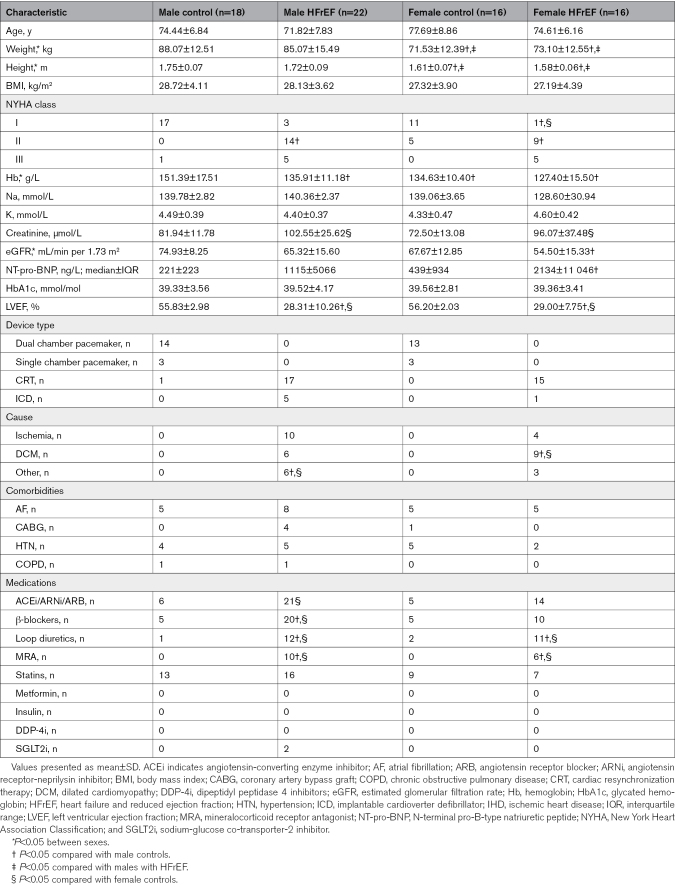
Clinical Characteristics for Men and Women With and Without HFrEF

### Sex Influences Muscle Transcriptome in Patients With HFrEF

**Figure 1.**
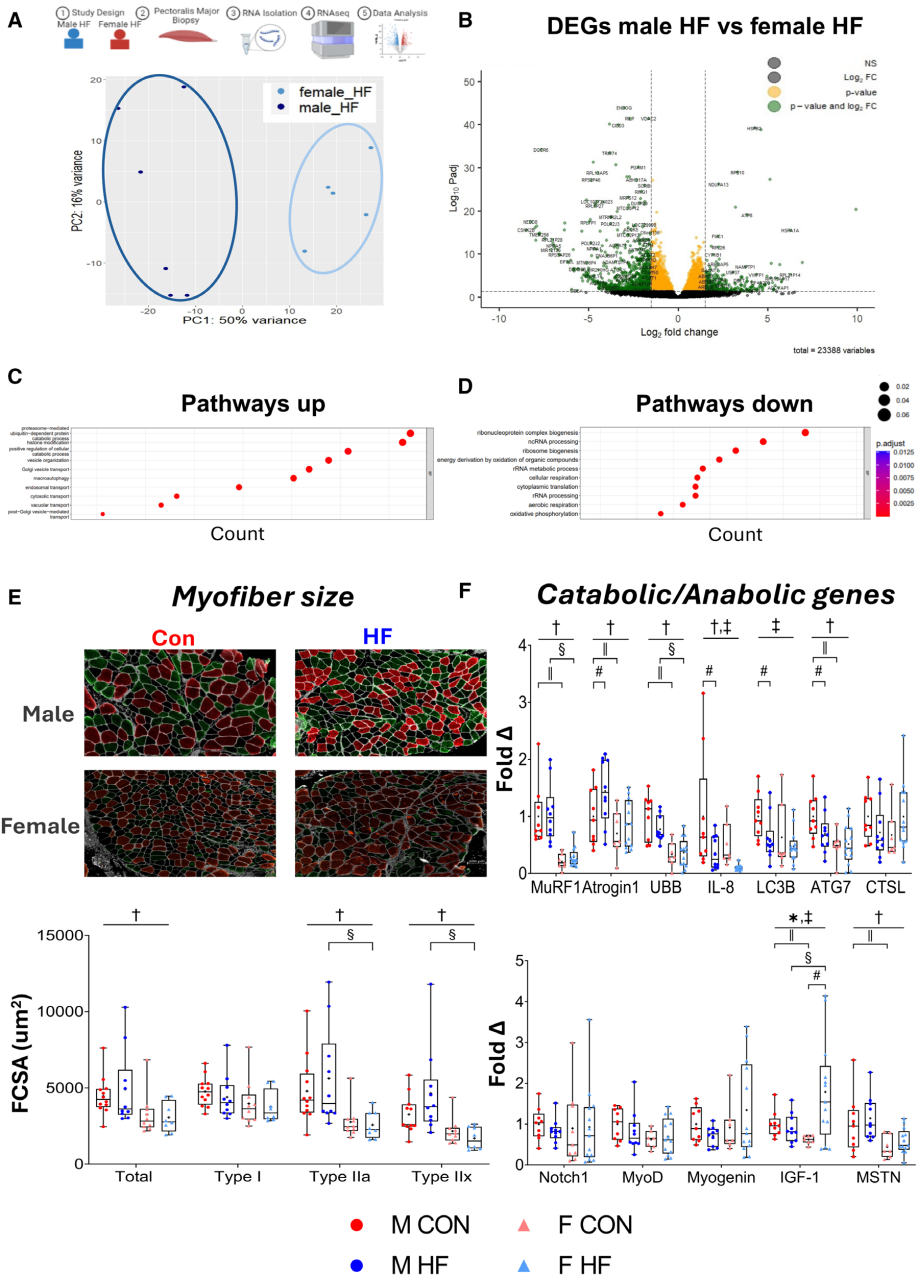
**Influence of sex on skeletal muscle transcriptome and myofiber atrophy. A**, Schematic of RNA-sequencing protocol for analysis between male (n=6) vs female (n=5) patients with heart failure and reduced ejection fraction (HFrEF) with principle component (PC) analysis plot showing HFrEF with man and woman are clustered into 2 distinct groups. **B**, DESeq2 analysis revealed 5629 differentially expressed genes (DEGs) were found when men were compared with women (green dots show the most significant DEGs [log2-fold change >1.5 or <−1.5] and yellow dots show smaller DEGs [log2-fold change >−1.5 or <1.5]) or <1.5])with significance threshold set at adjusted *P*<0.05 (Padj) corrected using Benjamini and Hochberg method. The Gene Ontology analysis of biological process with the top-10 terms by significance (adjusted *P*<0.05) for the upregulated (**C**) and downregulated (**D**) DEGs, as plotted by the gene count of the identified term. **E**, Representative images of pectoralis major skeletal muscle sections for males (M) including controls (CON; n=12) or HFrEF (n=10) and females (F) including CON (n=10) and HFrEF (n=8) with sections stained for fiber types (type I, red; type IIa, green; and type IIx, unstained/black) and quantified as fiber cross-sectional area (FCSA). **F**, mRNA expression (relative fold change) of atrophic, myogenic, and anabolic markers in male (CON: n=9; HFrEF: n=10) and female (CON: n=7; heart failure [HF]: n=13) patients. Data are presented as min-max box plots with median and quartile ranges denoted by the box, the mean denoted by the + symbol, and individual points plotted. ^*^P<0.05 denotes interaction between sex and HFrEF, ^†^*P*<0.05 denotes effect of sex, ^‡^*P*<0.05 denotes effect of HFrEF, ^§^*P*<0.05 denotes between-sex difference in HFrEF (ANOVA post hoc), ^‖^*P*<0.05 denotes between-sex difference in CON (ANOVA post hoc), and ^#^*P*<0.05 denotes within-sex difference (unpaired *t* test). ATG7 indicates autophagy-related protein 7; atrogin-1, F-box only protein 32; CTSL, cathepsin-L; IGF1, insulin-like growth factor-1; IL-8, interleukin-8; LC3B, microtubule-associated protein light chain 3; MSTN, myostatin; MuRF-1, muscle RING finger 1; MyoD, myoblast determination protein 1; Notch1, neurogenic locus notch homolog protein 1; and UBB, ubiquitin B.

Little is known about whether sex influences the muscle transcriptome in patients with HFrEF. To investigate this, we first conducted high-throughput unbiased RNA-sequencing (RNA-Seq) in a subcohort of males (control: n=5; HFrEF: n=6) and females (control: n=5; HFrEF: n=5) with and without HFrEF that had no differences in age, body mass index, comorbidities, or medications (Table 2). Principal component analysis plot analysis identified HFrEF with men and women as distinct groups by variance (Figure 1A). This was confirmed through DESeq2 analysis where 5629 differentially expressed genes (DEGs) were identified between sexes from a total of 23338 analyzed (Figure 1B). To further explore these differences, the DEGs were split by log2-fold change into those upregulated (n=2710) and downregulated (n=2919) in men compared with women followed by pathway enrichment analysis using the Gene Ontology database. This approach identified 256 terms downregulated and 799 terms upregulated in men compared with women with HFrEF, with the top-10 biological processes upregulated related to procatabolic processes such as the ubiquitin-proteasome system and autophagy that are key regulators of muscle atrophy (Figure 1C). In contrast, the top-10 biological processes downregulated in men compared with women were related to genes involved in mitochondrial oxidative phosphorylation (Table S1) and ribosome biogenesis that is involved in muscle fatigue and growth, respectively (Figure 1D). Additional terms related to metabolism, myogenesis, mTOR signaling, angiogenesis, and inflammation were also identified (Table S2).

**Table 2.**
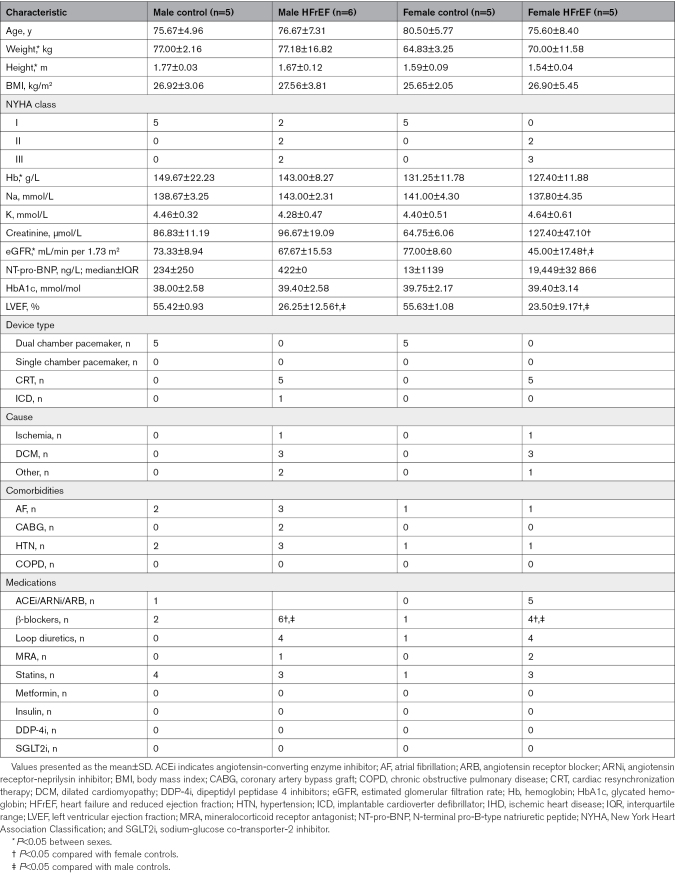
Clinical Characteristics for a Subset of Men and Women With and Without HFrEF That Underwent RNA-Sequencing

**Figure 2.**
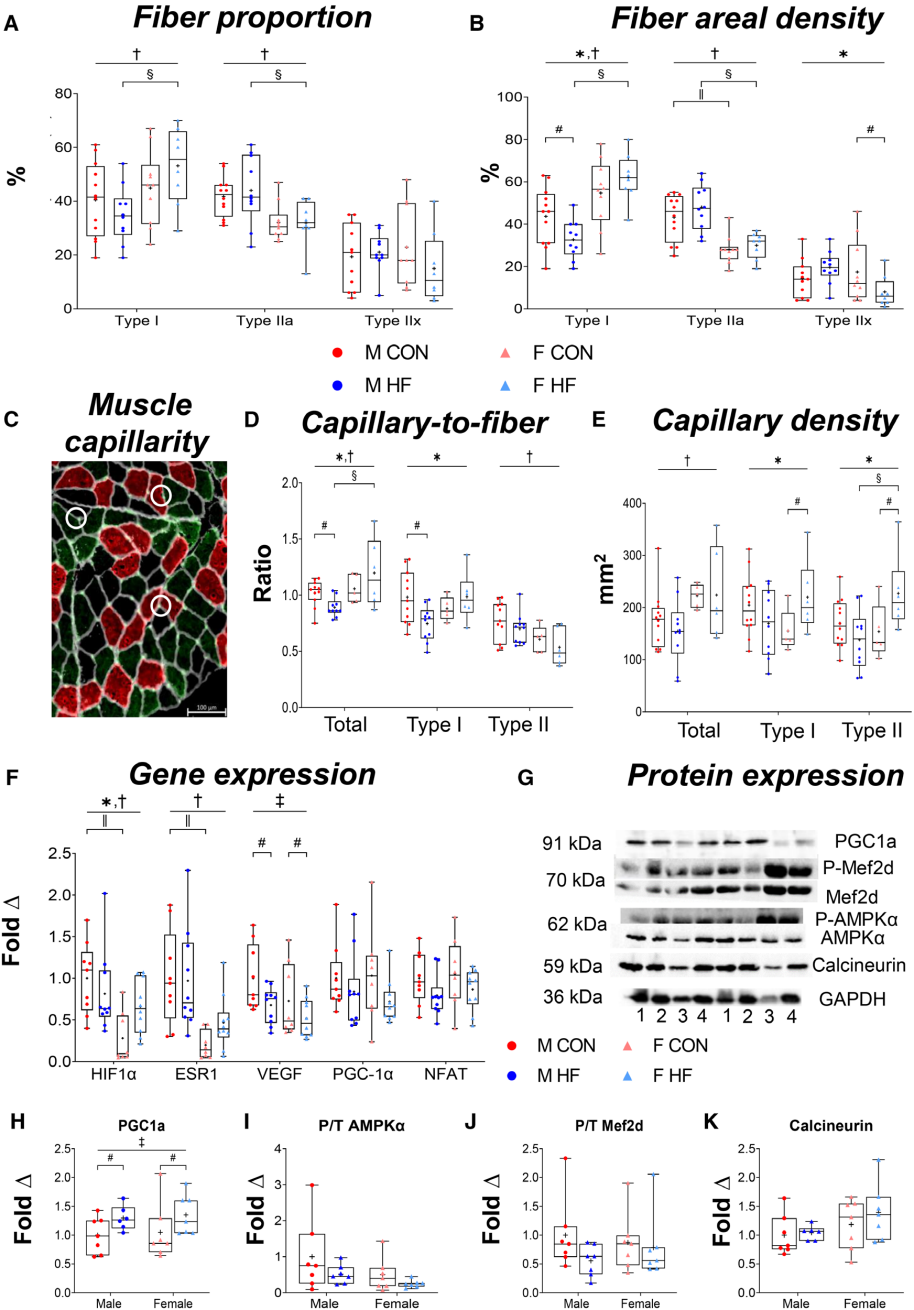
**Influence of sex on skeletal muscle phenotype and capillarity.** Muscle phenotype assessing fiber type proportion (**A**) and fiber areal density (**B**) including males (M) control (CON; n=12) or heart failure with reduced ejection fraction (HFrEF; n=10) and females (F; CON; n=5) or HFrEF (n=6). Representative stained muscle capillarity sections (white circles; **C**) with quantification of total, type I–, and type II–specific capillary-to-fiber (**D**) and capillary density (**E**). **F**, mRNA expression of molecular regulators of fiber phenotype in males (CON: n=9; HFrEF: n=10) and females (CON: n=8; HFrEF: n=10). **G**, Representative immunoblots in males (CON: n=7; HFrEF: n=7) and females (CON: n=7; HFrEF: n=7) with GAPDH (glyceraldehyde 3-phosphate dehydrogenase) used as loading control, with samples denoted by 1: male CON; 2: HFrEF with man; 3: female CON; and 4: HFrEF with woman. Quantified protein expression for total PGC1α (peroxisome proliferator-activated receptor-gamma coactivator 1 alpha; **H**), phosphorylated to total AMPK (AMP-activated protein kinase; **I**), phosphorylated to total MEF2D (myocyte enhancer factor 2D; **J**), and total calcineurin (**K**). Data are presented as min-max box plots with median and quartile ranges denoted by the box, the mean denoted by the + symbol, and individual points plotted. ^*^P<0.05 denotes interaction between sex and HFrEF, ^†^*P*<0.05 denotes effect of sex, ^‡^*P*<0.05 denotes effect of HFrEF, ^§^*P*<0.05 denotes between-sex difference in HFrEF (ANOVA post hoc), ^‖^*P*<0.05 denotes between-sex difference in CON (ANOVA post hoc), and ^#^*P*<0.05 denotes within-sex difference (unpaired *t* test). HF indicates heart failure.

In contrast to sex differences in HFrEF, a comparison of the transcriptome between the male and female control groups showed limited changes: only 39 DEGs were identified (Figures S1 and S2). We next explored HFrEF-specific effects on the muscle transcriptome within men or women. This analysis showed clear differences, with 2429 DEGs identified between male control versus HFrEF with men and 4126 DEGs identified between female control versus HFrEF with women (Figures S1 and S2).

In summary, these data indicate that a unique muscle transcriptome signature is present between men and women with HFrEF that may influence muscle pathology, which could not be explained by baseline sex differences.

### Sex Impacts Myofiber Atrophy in HFrEF

To strengthen our RNA-Seq analysis that sex may influence myofiber atrophy between men and women with HFrEF, we next quantified structural differences in sectioned and stained muscle biopsies collected from male and female patients with HFrEF and included age-matched controls. Representative muscle samples are presented in Figure 1E. There was no significant interaction between sex and HFrEF (*P*>0.05); however, an effect of sex was present such that women had lower mean fiber size compared with men (*P*<0.001; Figure 1E), and this occurred in a type II–specific manner (*P*<0.05; Figure 1E).

Next, targeted quantitative real time polymerase chain reaction was used to quantify the expression of catabolic and anabolic genes involved in muscle atrophy (Figure 1F), which strengthened our hypothesis that changes in myofiber size regulation occurred between men and women with and without HFrEF. An effect of sex was present for all catabolic genes related to the ubiquitin-proteasome system (*P*<0.05; Figure 1F), with lower expression in women compared with men for *MuRF-1* (*P*<0.001), *MAFBx*/*Atrogin-1* (*P*=0.008), *UBB* (*P*<0.001), and *IL* (interleukin)*-8* (*P*=0.044) and also for the autophagy gene *ATG7* (*P*=0.004). However, HFrEF was associated with a decrease in *LC3B* expression compared with controls (*P*=0.047; Figure 1F), indicating potential disease–specific inhibition of autophagy as previously reported in rodents.^22^

As inhibition of anabolic signaling and disturbed myogenic homeostasis can also contribute to atrophy,^16^ and our RNA-Seq analysis identified several terms related to these processes, we next explored changes in key myogenic regulators, *Notch1*, *MyoD*, and *myogenin*, but found few differences (*P*>0.05; Figure 1F). However, an interaction between sex and HFrEF was observed for the proanabolic factor *IGF1* (*P*=0.016; Figure 1F) such that women with HFrEF showed higher and men with HFrEF showed lower expression compared with controls. There was also an effect of sex (*P*=0.001) on the negative regulator of anabolic signaling *MSTN*, with increased expression in men with HFrEF compared with women with or without HFrEF (*P*=0.049 or *P*=0.029; respectively; Figure 1F).

In summary, these data indicate that atrophic signaling related to the proteasome, autophagy, and myostatin signaling is lower in women than men, whereas the proanabolic factor *IGF1* shows a sex-specific effect in HFrEF.

### Sex Regulates Muscle Fiber Type and Capillarity in HFrEF

In addition to the atrophy, muscle pathology in HFrEF is further characterized by early onset fatigue due to shifts in oxidative-to-glycolytic fiber types and reductions in muscle capillarity.^16^ To explore whether sex influences these characteristics, muscle biopsies were further examined to quantify fiber type of the oxidative type I and more glycolytic type IIa and type IIx fibers (Figure 1E). Whereas no interaction between sex and HFrEF was found for fiber type (*P*>0.05; Figure 2A), an effect of sex was found such that women showed higher type I and lower type IIa fibers compared with men (*P*=0.038; Figure 2A). However, an interaction between sex and HFrEF was found for fiber areal density including for type I (*P*=0.034) and type IIx (*P*=0.033) fibers (Figure 2B), whereas a further effect of sex was found for type IIa fibers (*P*<0.001; Figure 2B), both of which likely influence muscle atrophy between sexes.

We next examined whether muscle capillarity was different between sexes by quantifying both total and fiber-type–specific measures (Figure 2C). There was an interaction between sex and HFrEF for total capillary-to-fiber ratio (*P*=0.034; Figure 2D), and this occurred in a type I–specific manner (*P*=0.026; Figure 2D). A sex effect (*P*=0.008) for total and type II capillary-to-fiber ratio was also found, with post hoc analysis revealing between-group differences in capillary-to-fiber between women and men with HFrEF (*P*=0.005; Figure 2D). Complementary measures of muscle capillary density were also quantified in our muscle samples to strengthen interpretation (Figure 2E), and this revealed an interaction between sex and HFrEF for capillary density for type I and type II fibers (*P*=0.035 and *P*=0.012, respectively; Figure 2E), whereas an effect of sex (*P*=0.016) showed higher capillary density in women compared with men (Figure 2E).

Together, our structural imaging data indicate that while women generally have lower myofiber size and more type I fibers than men, a sex-specific effect in HFrEF is apparently related to fiber areal density and muscle capillarity.

### Sex Influences Molecular Signaling Coordinating Muscle Phenotype in HFrEF

We next explored underlying molecular pathways that could explain our sex-specific differences in muscle remodeling. We first explored molecular pathways controlling fiber phenotype, including PGC1α, HIF-1α, ESR1, NFAT, AMPK, MEF2, VEGF, and calcineurin.^23^ For mRNA expression, an interaction of sex and HFrEF was present for *HIF-1α* expression (*P*=0.040; Figure 2F), and there was an effect of sex with higher expression in men compared with women (*P*=0.002; Figure 2F). An effect of sex was also found for *ESR1* (*P*<0.001), with reduced expression in female controls compared with males (control: *P*=0.006; HFrEF: *P*=0.007; Figure 2F). Furthermore, an effect of HFrEF was found for *VEGF*, with lower expression in disease compared with controls (*P*=0.019 Figure 2F). No differences were found between men and women for *PGC1α* and *NFAT* gene expression (*P*>0.05; Figure 2F).

Next, we probed the protein content (both phosphorylated and total) of specific targets to reinforce our mRNA data (Figure 2G). Although no interaction between sex and HFrEF was detected for all proteins, there was an effect of HFrEF on PGC1α protein levels, with higher expression in patients with HFrEF compared with controls (*P*=0.043; Figure 2H). Although no effects on phosphorylated to total AMPK levels were found (Figure 2I), there was an effect of sex (*P*=0.035) for total AMPK expression with higher such that women showed higher expression compared with men. No further effects were found for MEF2D (Figure 2J) or calcineurin (Figure 2K) expression.

In summary, these data reveal that HFrEF sex-specific differences in the molecular regulators of muscle remodeling were present for *HIF-1α*, with further sex differences for *ESR1* and total AMPK expression and HFrEF differences apparent for *VEGF* and PGC1α.

### Sex Differences in Systemic Proinflammatory Cytokines in HFrEF

**Figure 3.**
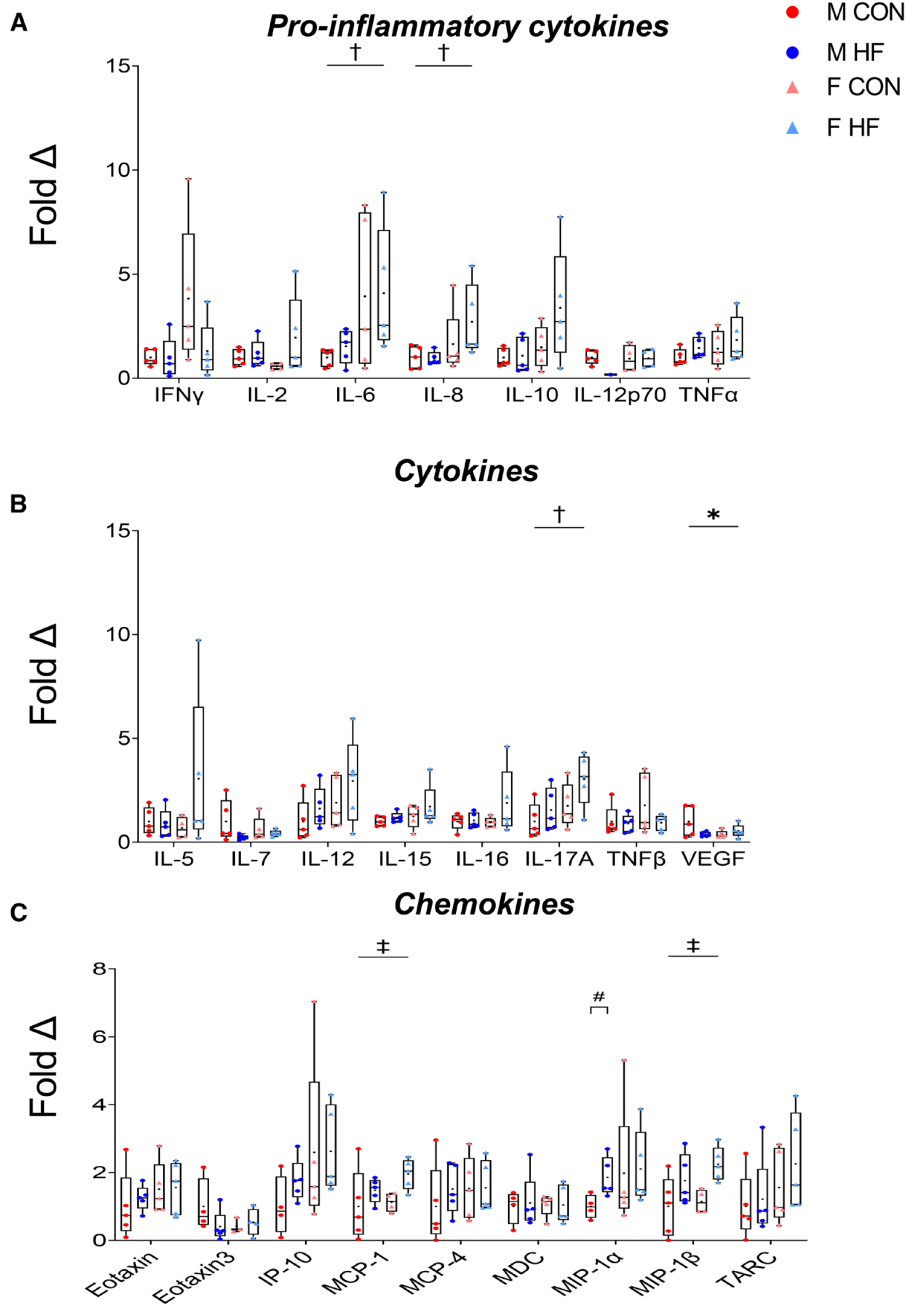
**Influence of sex of circulating inflammatory factors in serum.** Analysis of systemic proinflammatory cytokines (**A**), cytokines (**B**), and chemokines (**C**) in serum collected from men (M) and women (F), either control (CON) or heart failure with reduced ejection fraction (HFrEF; n=5 per group). Data are presented as minimum-maximum box plots with median and quartile ranges denoted by the box, the mean denoted by the + symbol, and individual points plotted. ^*^P<0.05 denotes interaction between sex and HFrEF, ^†^*P*<0.05 denotes effect of sex, ^‡^*P*<0.05 denotes effect of HFrEF, and ^#^*P*<0.05 denotes within-sex difference (unpaired *t* test). HF indicates heart failure; IFN, interferon; IL, interleukin; IP-10, interferon-γ-inducible protein 10; MCP, monocyte chemoattractant protein; MDC, C-C motif chemokine 22; MIP, macrophage inflammatory protein; TARC, CCL17/CC motif chemokine ligand 17; TNF, tumor necrosis factor; and VEGF, vascular endothelial growth factor.

At the systemic level, proteomics previously revealed sex differences in circulating proteins in HFrEF,^24^ but this has not been explored as a potential upstream factor influencing muscle pathology. To identify whether systemic factors are distinct between sexes and whether these were associated with muscle pathology severity, serum was collected to assess inflammatory profiles (Figure 3; Table S3). While there was no interaction between sex and HFrEF for all inflammatory cytokines assessed (Figure 3), there was an effect of sex for the atrophy-related cytokine IL-6 (*P*=0.024) and IL-8 (*P*=0.045), with women having higher concentrations relative to men (Figure 3A). Of the other cytokines analyzed, an interaction between sex and HFrEF was observed for VEGF (*P*=0.049; Figure 3B), suggesting a potential dual role at the systemic and local muscle level (eg, Figure 2F). An effect of sex was present for IL-17A (*P*=0.031), with women with HFrEF having higher concentrations compared with male controls (*P*=0.035; Figure 3B). An effect of HFrEF was also present in MCP (monocyte chemoattractant protein)*-1* (*P*=0.040) and MIP (macrophage inflammatory protein)*-1β* (*P*=0.009; Figure 3C); otherwise, no other effects were detected for remaining cytokines or chemokines.

In summary, our systemic inflammatory markers were mostly limited to sex differences (higher in women than men) or HFrEF differences (higher in HFrEF compared with controls); however, a specific sex difference in HFrEF was found for circulating VEGF.

## DISCUSSION

This study comprehensively investigated sex differences in skeletal muscle pathology between male and female patients with HFrEF. Overall, by combining muscle transcriptome sequencing, structural myofiber imaging, molecular signaling phenotyping, and systemic inflammatory profiling, the major findings of this study showed the following.

1. Distinct and widespread differences in the muscle transcriptome were found between men and women with HFrEF, which were specific to catabolic and mitochondrial terms.
2. Women with HFrEF showed lower muscle atrophic signaling than men with HFrEF, including reduced proteasome, autophagy, and myostatin expression, but higher proanabolic IGF1 expression.
3. Structurally, compared with men, women with HFrEF were characterized by a proendurance muscle phenotype as characterized by higher muscle capillarity alongside a smaller but greater number of fatigue-resistant type I fibers.
4. Underlying differences in molecular regulators of muscle phenotype were detected between sexes including for HIF-1α, ESR1, VEGF, *and* PGC1α.
5. Limited differences in circulating serum proinflammatory cytokines were found between sexes, but VEGF was higher in women compared with men with HFrEF.
6. Effects of sex on muscle pathology in HFrEF should be interpreted with caution unless appropriate sex-matched controls are included.

### Sex Differences in Muscle Atrophy and Proteolytic Activation

Earlier studies that compared muscle atrophy between men and women with HFrEF via histological assessment have reported limited differences^13,14^ and no assessed changes in underlying molecular pathways. However, more recent evidence using noninvasive imaging indicates that men with HFrEF may develop more muscle wasting than women with HFrEF.^25^ Here, we performed multiple assessments for muscle atrophy related to structural imaging, RNA-Seq, and targeted gene/protein expression, to verify atrophy and related signaling pathways. Comparison of the muscle transcriptome between men and women with HFrEF revealed >5000 DEGs, which indicated a widely divergent profile between sexes in line with reports from healthy individuals.^26–28^ Enrichment analysis further identified that key biological processes for catabolic processes were upregulated in men compared with women with HFrEF. Furthermore, these observations were strengthened when we validated (via quantitative real time polymerase chain reaction) that expressions of key proteolytic pathways related to the ubiquitin proteasome, autophagy, and myostatin were higher in men than women. Past evidence in skeletal muscle from healthy people indicates a lower basal expression of genes related to ubiquitin-dependent protein catabolism is present in women compared with men.^29^ These findings provide new evidence in patients with HFrEF that sex influences muscle atrophic pathway expression, which, in turn, could influence the muscle wasting profile.

However, no interaction was found between sex and HFrEF for myofiber size, and, instead, women generally showed lower myofiber sizes compared with men, which is in line with findings from healthy people.^30–32^ However, when we normalized our data to fiber areal density, women as a whole showed higher type I fiber size relative to men.^32^ In addition, an interaction between sex and HFrEF was found for *IGF1* expression such that levels were highest in women but lowest in men with HFrEF. IGF1 promotes both muscle protein synthesis and myogenesis,^33–35^ and its expression is reported to be decreased and associated with muscle atrophy in men with HFrEF.^36^ Although there is little evidence at this point to explain why IGF1 would be higher in women with HFrEF, some data from rodent models have shown a woman-specific effect in muscle.^37^ This includes a link between IGF1 levels and higher muscle capillarity.^37^ Intriguingly, overexpression of muscle-specific IGF1 in transgenic mice induced with HFrEF was shown to prevent muscle atrophy in line with reducing ubiquitin-proteasome system and atrogene expression, in addition to attenuating capillary rarefaction.^38^ Our current data in humans, therefore, closely align with these murine experiments and potentially explain a link between higher IGF1 expression, greater muscle capillarity, and lower atrophic signaling observed in women with HFrEF.

Moreover, in line with recent evidence in patients,^17^ we did not find obvious sex differences in circulating inflammatory markers in our HFrEF cohort. Many of these markers can increase myocyte injury, oxidative stress, and activate NF-κΒ (nuclear factor kappa B) signaling to induce muscle wasting.^39^ For example, past studies in men with HFrEF have shown a link between elevated levels of specific proinflammatory cytokines, such as IL-6, TNF (tumor necrosis factor) α, IL1β, and greater muscle pathology.^16,25,39^ However, to date, this link in women with HFrEF has not been well addressed. Our data did not find a clear HFrEF-specific sex effect, but rather it identified baseline sex or disease differences for both established (eg, IL-6, IL-8) and less established (IL17A, MCP1, and MIP-1β) circulating inflammatory factors. Albeit in a modest sample size, the current study provides initial evidence to indicate that cytokines may not be a primary upstream mechanism influencing muscle pathology between sexes in HFrEF, but future experiments are required to test this hypothesis further.

Together, therefore, these findings suggest that dysregulated muscle atrophy may not always underlie worse symptoms in women compared with men with HFrEF.^9^ Interestingly, compared with controls, a fiber type shift toward type II fibers in patients with HFrEF was not observed. This is inconsistent with the current literature but is probably explained by the use of pectoralis major biopsies in this study, whereas the majority of previous studies used leg biopsies. There are subtle differences between these muscles in terms of metabolic properties, which may underlie any observed differences. For example, the vastus lateralis has a higher type I oxidative capacity than pectoralis major^40,41^; however, in general, these muscles show similar fiber type compositions, basal protein synthesis rates,^42^ and overall similar signs of HF-induced muscle pathology.^41^ Nevertheless, other potential factors causing differential responses between these muscles could be related to variations in muscle activity patterns; for example, the upper limb muscles are unlikely impacted to the same degree as the lower limbs by confounding factors such as disuse/inactivity, aging, or arthritis.^41,43,44^

### Sex Differences in Muscle Phenotype and Capillarity

Past studies have shown that men with HFrEF have lower muscle capillary density and a higher number of type II fibers compared with male controls,^16^ but evidence from women is lacking.^13,14,45,46^ Up until now, changes related to fiber type reported between sexes in HFrEF have remained unclear,^13,14,46^ whereas the influence of sex on the underlying molecular regulators of fiber phenotype remained poorly addressed. Here, we show new data that indicate women compared with men with HFrEF present with a pro-endurance (or oxidative) muscle phenotype, demonstrating more type I fibers and higher muscle capillarity. Although muscle capillarity tended to be the highest in women with HFrEF compared with all other groups, future experiments should be performed in a larger sample size to strengthen this observation. Nevertheless, this trend follows data from healthy humans.^47,48^ Moreover, we further identified a distinct interaction between sex and HFrEF such that women with HFrEF showed a unique increase in these pro-endurance features compared with control females, whereas men with HFrEF showed a decrease relative to male controls. This finding was reinforced by our RNA-Seq data, which indicated that various terms related to angiogenesis and mitochondria were higher in women compared with men with HFrEF.

Whether this unique muscular phenotype in women with HFrEF represents a compensatory mechanism to maintain oxidative capacity and limit pathological progression (eg, due to the higher baseline pro-oxidative phenotype) remains unclear.^14,49^ To investigate this further, we explored potential underlying molecular signals known to be involved in regulating muscle phenotype. Muscle expressions of HIF-1α,^50–52^ ESR1,^53^ VEGF,^54^ and PGC1α,^54^ all known to be important regulators of a pro-oxidative muscle phenotype related to fiber type transitions, mitochondrial biogenesis, and angiogenesis, were influenced by either sex, HFrEF, or both. These changes may help to explain why some of our structural changes related to muscle capillarity, fiber type, and myofiber size/atrophy signaling occurred, but the current study is unable to provide definitive conclusions. Interestingly, PGC1α protein expression was increased in HFrEF for both sexes respective to controls. This corroborates previous data, which suggested that this may be a compensatory response to balance atrogene expression.^55^ However, past studies in human and animal models indicate decreased or no change in PGC1α skeletal muscle expression^15,56,57^; therefore, the current literature remains inconsistent. This may relate to antibody specificity and also posttranslational modifications of PGC1α. Further studies are, therefore, warranted to explore the role of PGC1α in muscle pathology in HFrEF.

Finally, we identified an interaction between sex and HFrEF for circulating concentrations of VEGF in the serum. Whether this could act as an upstream mediator influencing muscle phenotype or pathology in HFrEF remains to be determined although some evidence shows that increased circulating VEGF levels are associated with positive outcomes in HFrEF such as increased angiogenesis and platelet activity.^54^ Taken together, these data suggest that compared with men, women with HFrEF develop a more fatigue-resistant, pro-oxidative muscle phenotype that may protect against muscle pathology.

### Limitations

Our findings may not be applicable to the general HFrEF population, as it was limited to a single center and a modest sample size. While we included consecutive patients with HFrEF, the cohort included only people with HFrEF indicated for device therapy. This introduces a selection bias in that we did not include people with less severe left ventricle dysfunction or HF with mildly reduced ejection fraction. Future confirmatory work would need to include patients with HF with a range of left ventricle impairment and exercise limitations.

For controls, we excluded people with signs and symptoms of HF and those fulfilling echocardiography criteria for HF with preserved ejection fraction from the analysis. Despite this, NT-pro-BNP (N-terminal prohormone of brain natriuretic peptide) levels in controls were elevated and, as previously observed,^58^ were higher in women than men despite similar clinical and echocardiographic variables and treatment. NT-pro-BNP elevation has particularly poor specificity in older women^59^ such that the need for a pacemaker might have contributed to this difference in the absence of HF. This, in addition to the presence of comorbidities in the control group, may have attenuated larger differences in muscle pathology being detected between the control and HFrEF groups. We also did not have measures of exercise intolerance or quality of life scores, meaning that we were unable to identify how muscle differences between groups related to functional outcomes.

Due to limitations in muscle and serum availability, we were unable to perform all analyses on all samples, leading to variation in the number of samples used for analyses. However, accessing muscle biopsies in humans represents an approach which is technically and ethically challenging. Most patients are not willing to undergo the perceived risks associated with muscle biopsy. We also sampled a single region of muscle and assumed that this represented the larger muscle mass, but further whole-body imaging would help confirm this. Furthermore, the gene expression changes identified with the RNA-Seq do not necessarily correspond to changes at the protein level (Tables S4 and S5), which should be taken into consideration.

### Conclusions

This study suggests sex differences affect muscle pathology in patients with HFrEF, with men generally showing greater muscle abnormalities compared with women. Sex differences included changes related to muscle transcriptome, myofiber size and phenotype, capillarity, molecular signaling, and circulating factors. Taken together, these preliminary data question whether muscle pathology is a primary contributor to the greater symptoms reported in women compared with men with HFrEF. Additional studies are required to further confirm and explore this important gap in knowledge.

## ARTICLE INFORMATION

### Acknowledgments

The authors acknowledge the technical support and facilities provided by the University of Manchester for the serum cytokine analysis. The research was carried out at the National Institute for Health and Care Research Leeds Biomedical Research Centre.

### Sources of Funding

Dr Bowen received funding from the Medical Research Council (United Kingdom; grant MR/S025472/1), Heart Research UK (grant TRP16/19), and the British Heart Foundation (BHF; grant PG/21/10547). Dr Cheng was supported by the BHF Mautner Career Development Fellowship.

### Disclosures

None.

### Supplemental Material

Supplemental Methods

Tables S1–S5

Figures S1 and S2

References 60 and 61

## Supplementary Material


