# Supplementary material for: Sex Differences in Skeletal Muscle Pathology in Patients With Heart Failure and Reduced Ejection Fraction
Source: Circ Heart Fail. 2024 Oct 9;17(10):e011471. doi: 10.1161/CIRCHEARTFAILURE.123.011471 (PMC11472905; doi:10.1161/CIRCHEARTFAILURE.123.011471)
Supplement: Supplementary file 1 [file hhf-17-e011471-s001.pdf]

SUPPLEMENTAL MATERIAL

Supplemental Methods

Patients

Male (n = 22) and female (n = 16) patients with established HFrEF, who had persistent symptoms and a left ventricular ejection fraction (LVEF) < 40 % (as confirmed by echocardiography despite having receiving at least three months of guideline-directed medical therapy; in line with international guidelines)[21] undergoing routine cardiac implantable electronic device (CIED) implantation at Leeds Teaching Hospitals were approached to take part in this study. Patients with no evidence of heart failure (LVEF > 40 %) but requiring CIED implantation served as controls (n = 18 male, n = 16 female). The present analysis is based upon samples collected prior to and following the licensing of SGLT2i for HFrEF, allowing us to compare similar populations who would be indicated according to contemporary heart failure guidelines. All participants provided written informed consent and all procedures were conducted in accordance with the Declaration of Helsinki after receiving local institute ethical approval (11/YH/0291). During CIED implantation (between 2015 - 2020), skeletal muscle biopsies from the *pectoralis major* were collected from each patient, frozen in liquid nitrogen, and stored at -80 °C. Subsequent muscle analyses were then performed including structural imaging, transcriptomics, gene and protein expression, and serum profiling. All tissue samples were blinded by a unique ID number and subsequently analysed in a randomized order.

Histological Analysis

Samples were cut into 10 µm thick sections. Slides were initially blocked with 5 % goat serum in phosphate-buffered saline (PBS) for one hour. Staining for muscle fibers were conducted using the primary antibodies BA-D5 (Type I, 14.8:250 µl in 5 % goat serum) and SC-71 (Type IIa, 7.6:250 µl in 5 % goat serum). Secondary antibodies, IgG2b Alexa-Fluor 647 nm (Type I, 1:500 µl) and IgG Alexa-Fluor 488 nm (Type IIa, 1:500 µl), were added following the respective primary. Capillaries were stained using UEA I Fluorescein (5:200 µl in 5 % goat serum). Each antibody was applied for 1 hour with 3 x 5 minute washes using PBS-tween 0.05 % in between each antibody application. Fiber boundaries were stained using wheat germ agglutinin (WGA) Rhodamine for 10 minutes (1:1000 µl in PBS). Non-stained fibers were assumed to be type IIx fibers. Slides were imaged on a slide scanner (AxioScan Z.1, 20x magnification, Zeiss) and exported into Zen blue imaging software (Zen 3.2 Blue edition, Zeiss) to produce regions of interest with approximately 100 fibres. Analysis of regions of interest were performed using DTECT and Capillar software described elsewhere[60].

Gene Expression

RNA extraction was performed using the trizol-chloroform-isopropanol method following Trizol reagent solution user guide (ThermoFisher Scientific). RNA purification using the RNA clean and concentrate-5 kit (Zymo) was conducted following the kit protocol resulting in high quality RNA in 20 µl of RNase-free water, confirmed using a nanodrop. cDNA synthesis was conducted using the RT^2 First Strand kit (Qiagen) following kit protocols. PCR was performed using a Bio-Rad CFX 96 Thermocycler. Genes were run in triplicate for each sample. A mix of 10 µl SYBR Green (SYBR green Light Cycler 480 I master mix, Roche) 0.6 µl of lab validated primers containing both forward and reverse primers (Supplementary Table 4), 8.6 µl of Rnase free water and 0.8 µl of cDNA were pipetted into the well of each plate. Delta-Delta-CT method was then performed to calculate fold expression changes normalised to beta-actin.

RNA-sequencing

Non-biased RNA sequencing was performed using an aliquot of the RNA isolated for gene expression. A 20 ul Eppendorf containing 600 ng RNA for each sample was sent to Novogene (Cambridge, UK) for library preparation, library QC and sequencing (RNAseq). Raw files were processed in UNIX shell. Raw FASTQ files were assessed for quality and overrepresented sequences using FASTQC. Raw reads were trimmed using TRIM Galore package to remove low-quality reads, sequences below 20 base pairs and adapter sequences. A reference human genome was created using information from the Ensembl genome database (GRCh38.p14). STAR aligner was then used to align the trimmed sequences to the reference human genome. Once aligned, FeatureCounts package was used to count the number of sequence instances in the samples with a minimum requirement of 10 counts. Analysis including principal component analysis (PCA), differentially expressed gene (DEG) analysis (DESeq2 v1.40), gene ontology enrichment (ClusterProfiler v4.6.0) and pathway analyses (KEGG via The Database for Annotation, Visualisation, and Integrated Discovery 2021 and ReactomePA v1.42.0) were all conducted from the counts file in R v4.3.0 with a false discovery rate of $p < 0.05$ (p.adjusted) accepted as statistically significant and log2 fold change > 1.5 or < -1.5. As 10 controls samples were sequenced at a later date, the batch effects in the analyses were corrected using the ComBat tool available in the sva R package[61]. The raw data are available at ArrayExpress under the accession number E-MTAB-8531 and E-MTAB-14110, whereas the link to the RNAseq code is available via https://github.com/Cheng-CW/Than_etal_2024_HFrEF.git.

Western Blot

A 15-20 mg piece of pectoralis major tissue was homogenised in a tube with approximately 150 µl of radioimmunoprecipitation assay buffer (RIPA) containing 1 ml RIPA lysis buffer 10 x, 9ml of ddH2O and 1 tablet of Pierce protease and phosphatase inhibitor (ThermoFischer Scientific). Homogenate was then centrifuged at 4 °C for 10 minutes at 12,000 g and the supernatant transferred into a new tube. Protein quantification of homogenate was conducted using Pierce BCA protein assay kit (ThermoFisher Scientific). Standards were prepared according to kit protocol and 20 µl of each standard was pipetted into wells in duplicate. 2 µl of sample and 18 µl of DDH_2O were pipetted in duplicate into desired wells. BCA reagent was prepared in a 50:1 ratio and 180 µl added to each well. Plates were incubated in foil at 37 °C for 30 minutes and immediately quantified using VarioSkan Flash (ThermoFisher Scientific) at absorbance 570 nm. Protein quantification was calculated using the standard curve and absorbance values of the samples. Samples were then prepared for gel electrophoresis by combining sample with DDH_2O and a quarter total volume desired of Laemmli buffer 4x creating a volume with 2 µg/µl protein. Pre-cast gels (Bio-Rad mini protean precast gels) were run by loading 10 µl (20 µg) of protein and 4 µl of protein ladder into the final well. Transfer onto a nitrocellulose membrane was completed in a Bio-Rad transblot turbo transfer system. Ponceau S staining was conducted to verify transfer of proteins, then membranes were blocked in 5 % bovine serum albumin (BSA) for phosphorylated proteins or 5 % milk for 90 minutes. Primary antibodies were diluted in 5% BSA according to manufacturer's datasheets (Supplementary Table 5) and incubated overnight. Following 3 washes (5-10-5 min) in TBS-T20, secondary antibody was applied corresponding to primary antibody source in either 3% milk (if blocked in milk) or 5 % BSA (if blocked in BSA). Final washes in TBS-T20 were conducted and then membranes were imaged on an iBright CL-750 (Invitrogen) using Pierce ECL blotting substrate peroxide solution and luminol enhancer. Blots were then analysed in ImageJ and normalised to the loading control

Serum

Blood samples were collected through venepuncture and stored on ice before being centrifuged at 1,000 g for 10 minutes at 4 °C, separating the serum. 150 ul of serum was aliquoted for cytokine assay analysis. Cytokine and chemokine analysis was conducted using the V-PLEX human cytokine 30-plex kit (Mesoscale) following the three kit protocols (including alternate protocol 3). Plates were analysed with the MESO quickplex SQ 120 plate reader. Concentrations were then calculated using the absorbance measurements and the standard curve line equation. Of the 30 cytokines analysed, 6 were not present in a sufficient quantity

to reach threshold value for detection by the plate reader (GM-CSF, IL-1α, IL-1β, IL-4, IL-8(HA), IL-13).

Statistical Analysis

Data were analysed in Prism (GraphPad Prism 9 v9.4.1). Maurice Test of Sphericity was conducted to assess normality. Two-way ANOVAs were conducted to assess differences between condition and sex. Tukey's multiple comparison tests were conducted as part of the two-way ANOVA to assess differences between all groups. T-tests were conducted for within-group comparisons. Chi squared (X^2) tests were used to compare categorical clinical variables. Comparisons between controls of one sex to patients with HFrEF of the other sex (i.e., male controls vs female HFrEF or female controls vs male HFrEF) were not included as these were not directly relevant to the study aims and hypotheses. Statistical significance was accepted at $p < 0.05$ and data are presented as min-max box plots with median, quartile ranges and the mean displayed unless stated otherwise.

Supplemental Figures and Figure Legends

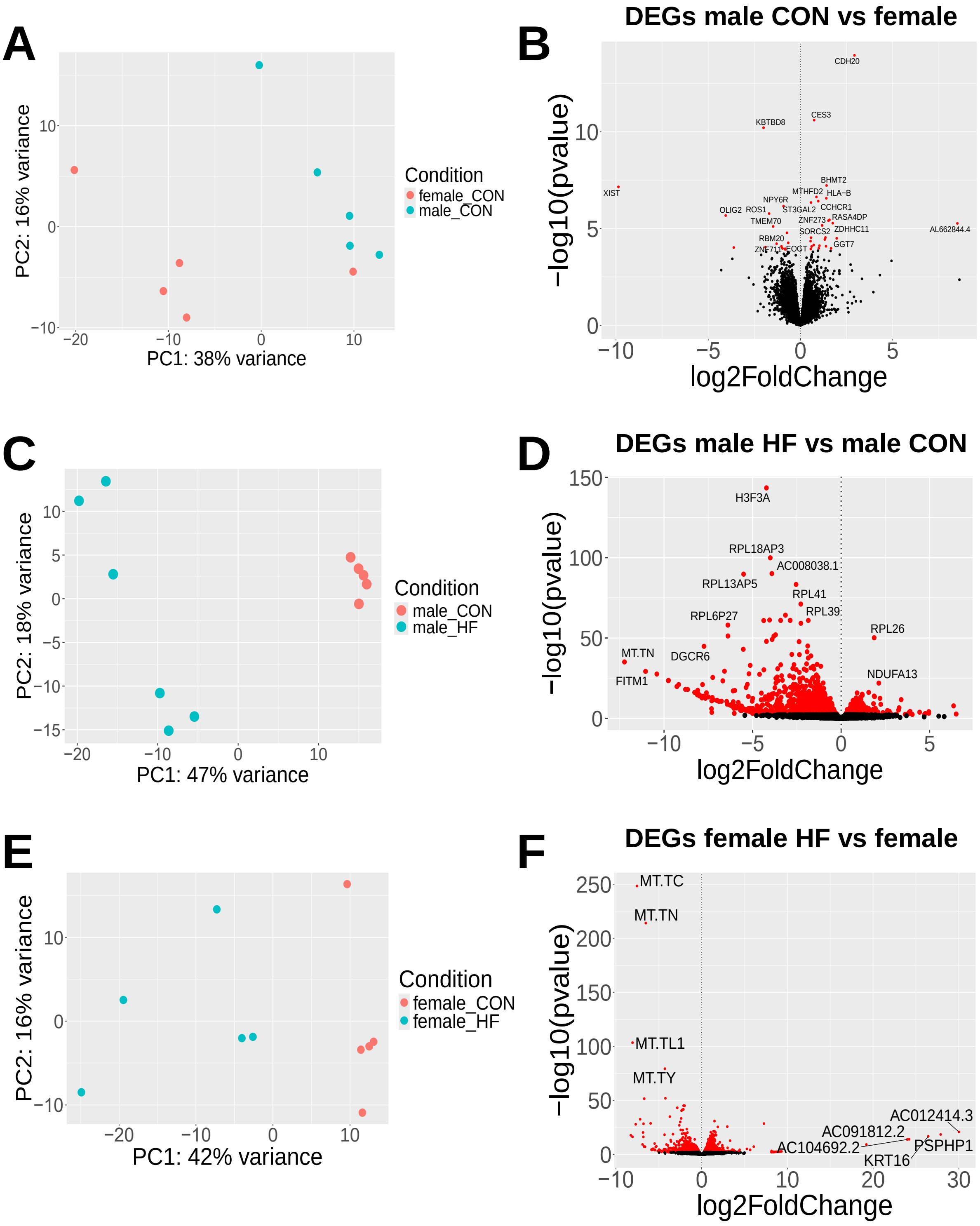

Figure S1

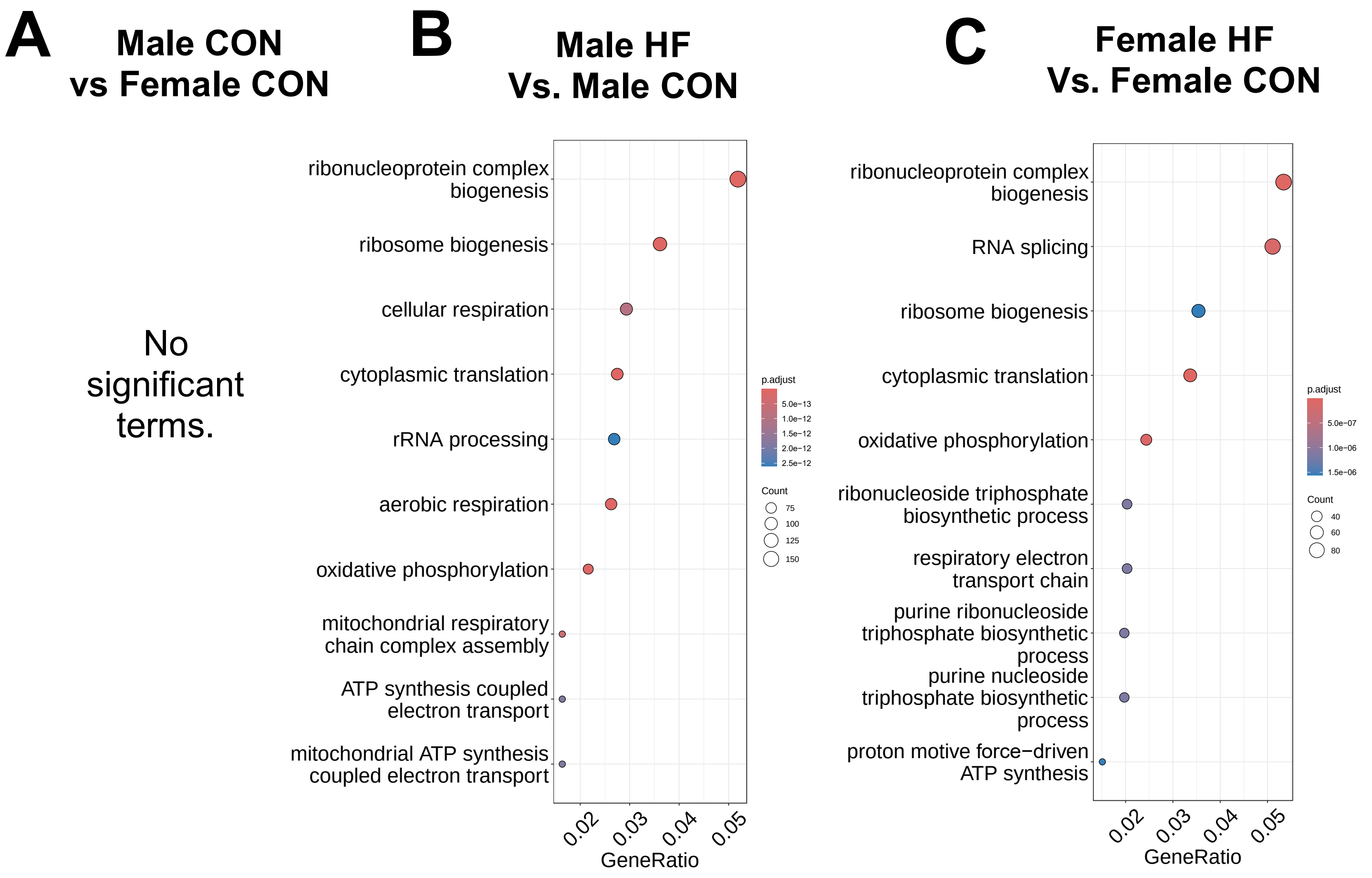
A
Male CON
vs Female CON
No significant terms.
B
Male HF
Vs. Male CON
ribonucleoprotein complex biogenesis
ribosome biogenesis
cellular respiration
cytoplasmic translation
rRNA processing
aerobic respiration
oxidative phosphorylation
mitochondrial respiratory chain complex assembly
ATP synthesis coupled electron transport
mitochondrial ATP synthesis coupled electron transport
0.02
0.03
0.04
0.05
GeneRatio
p.adjust
5.0e−13
1.0e−12
1.5e−12
2.0e−12
2.5e−12
Count
75
100
125
150
C
Female HF
Vs. Female CON
ribonucleoprotein complex biogenesis
RNA splicing
ribosome biogenesis
cytoplasmic translation
oxidative phosphorylation
ribonucleoside triphosphate biosynthetic process
respiratory electron transport chain
purine ribonucleoside triphosphate biosynthetic process
purine nucleoside triphosphate biosynthetic process
proton motive force−driven ATP synthesis
0.02
0.03
0.04
0.05
GeneRatio
p.adjust
5.0e−07
1.0e−06
1.5e−06
Count
40
60
80

Figure S2

Figure S1. RNA-seq analyses of 4 different groups; male controls (n = 5), female controls (n = 5), male patients with heart failure (HF) with reduced ejection fraction (HFrEF; n = 6) and female patients with HFrEF (n = 5). Analysis of male against female controls. (**A**) Principle component analysis (PCA) plot shows two clusters of individuals with PC1 explains 38 % variance and PC2 with 16 % variance. (**B**) DESeq2 analysis identified 39 differentially expressed genes (DEGs) between male and female controls. Red dots represent statistically significant dysregulated genes on adjusted *p value* < 0.05 corrected using Benjamini and Hochberg method. Analysis of male with HFrEF against male controls. (**C**) PCA plot shows two distinct groups separating male with HFrEF from controls. (**D**) DESeq2 analysis revealed 2429 DEGs based on adjusted *p value* < 0.05. Analysis of female with HFrEF against female controls. (**E**) Two groups of individuals clustered distinctly on the PCA plot with PC1 accounts for 42 % variance and PC2 with 16 % variance. (**F**) DESeq2 analysis identified 4126 DEGs based on adjusted *p value* < 0.05.

Figure S2. Gene ontology analysis showing the top 10 significant (adjusted *p* value < 0.05) pathways by gene ratio (the number of genes identified in the enriched term compared to the total number of identified differentially expressed genes; DEGs) for each group comparison: (**A**) Male vs female controls (CON), (**B**) Male heart failure (HF) with reduced ejection fraction (HFrEF) vs male CON and (C) Female HFrEF vs female CON.

Supplemental Tables

Table S1. List of significant genes identified in the mitochondrial oxidative phosphorylation biological process term which was downregulated in males compared to females with heart failure with reduced ejection fraction (HFrEF).

Gene	Log2 fold change	*p*-value
CHCHD10	-4.46018	2.39E-108
MTCO2P12	-2.77125	3.35E-15
ANTKMT	-2.29699	8.94E-12
NDUFA6	-2.10563	1.42E-23
NDUFA3	-2.05998	5.50E-12
ATP5F1D	-1.75982	2.23E-09
NDUFS3	-1.69399	8.89E-11
NDUFS8	-1.524	3.36E-09
NDUFS6	-1.50165	1.10E-06
MLXIPL	-1.32199	0.031638
COX5B	-1.3216	5.67E-06
NDUFB7	-1.31059	4.05E-05
SDHAF2	-1.23125	0.003448
COX7A1	-1.20726	0.00056
NDUFB11	-1.17351	0.000105
CHCHD2	-1.17213	5.25E-07
UQCRH	-1.04403	2.00E-07
COX7C	-1.04067	1.59E-05
COX6A2	-1.02875	0.000206
UQCRC1	-0.90561	2.56E-06
ND3	-0.90381	0.00015
NUPR1	-0.89846	0.026319
NDUFV1	-0.87033	0.000415
NDUFB10	-0.86959	0.000393
TAFAZZIN	-0.81994	0.00379
UQCR10	-0.81149	0.001844
UQCRFS1	-0.80745	0.000296
COX6B1	-0.79491	0.022481
NDUFB4	-0.75338	3.85E-06
ABCD1	-0.74677	0.028908
DNAJC30	-0.73685	0.025789
NDUFA8	-0.72958	0.002464
NDUFV3	-0.72771	0.006086

NDUFS5	-0.71618	0.048973
COX4I1	-0.71587	0.008718
NDUFAB1	-0.70529	0.011691
UQCRC2	-0.68447	0.006896
COX6C	-0.6843	0.020689
COX7A2	-0.6815	0.026467
UQCRQ	-0.67914	0.034895
ATP5F1E	-0.6697	0.035357
CYC1	-0.66882	0.005037
PPIF	-0.66783	0.010656
UQCC2	-0.65627	0.004753
COX5A	-0.64159	0.005882
NDUFA10	-0.61644	0.003302
STOML2	-0.6163	0.002731
ATP5MF	-0.60152	0.015679
ATP5PB	-0.60004	0.021799
NDUFB3	-0.58191	0.022223
SDHA	-0.58148	0.005153
NDUFC1	-0.55652	0.033349
SHMT2	-0.46369	0.007771

Table S2. Key terms identified outside of top 10 by significance related to skeletal muscle pathology in males compared to females with heart failure with reduced ejection fraction (HFrEF).

Analysis	GO	KEGG	Reactome
Downregulated DEGS (males vs females)	aerobic electron transport chain	Notch signalling pathway	Eukaryotic Translation Elongation
	ATP synthesis coupled electron transport	mTOR signalling pathway	Formation of a pool of free 40S subunits
	cellular amino acid metabolic process	Fatty acid degradation	The citric acid (TCA) cycle and respiratory electron transport
	TOR signalling	Insulin signalling pathway	mTORC1-mediated signalling
	negative regulation of ubiquitin protein ligase activity	VEGF signalling pathway	Cristae formation
		FoxO signalling pathway	
		TNF signalling pathway	
		HIF-1 signalling pathway	
Upregulated DEGS (males vs females)	protein polyubiquitination	TGF-beta signalling pathway	Antigen processing: Ubiquitination & Proteasome degradation
	transforming growth factor beta receptor signalling pathway	Ubiquitin mediated proteolysis	Signalling by TGFB family members
	regulation of macroautophagy	FoxO signalling pathway	Oncogenic MAPK signalling
	protein deubiquitylation	Autophagy - animal	Signalling by VEGF
	regulation of ubiquitin-dependent protein catabolic process	JAK-STAT signalling pathway	Toll Like Receptor 4 (TLR4) Cascade
	membrane fission	Apoptosis	
	mRNA catabolic process	NF-kappa B signalling pathway	
	positive regulation of cytokine production	p53 signalling pathway	
		mTOR signalling pathway	

Table S3. Concentrations of serum circulating factors (in pg/mL) in males and females with heart failure and reduced ejection fraction (HFrEF) and controls (CON)

Cytokine/Chemokine	Male CON (n=5)	Male HFrEF (n=5)	Female CON (n=5)	Female HFrEF (n=5)
IFNγ	5.68 ± 0.97	5.32 ± 2.51	21.75 ± 8.77	7.41 ± 3.51
IL-2	0.40 ± 0.07	0.46 ± 0.12	1.49 ± 1.25	0.78 ± 0.35
IL-6†	0.67 ± 0.13	1.03 ± 0.25	2.65 ± 1.13	2.75 ± 0.92
IL-8†	6.82 ± 1.65	6.51 ± 0.96	11.22 ± 4.88	18.48 ± 5.38
IL-10	0.23 ± 0.05	0.25 ± 0.09	0.35 ± 0.11	0.79 ± 0.29
IL-12p70	0.17 ± 0.02	0.03 ± 0	0.15 ± 0.05	0.16 ± 0.04
TNFα	1.18 ± 0.21	1.71 ± 0.27	1.68 ± 0.45	2.17 ± 0.59
IL-5	0.82 ± 0.24	0.71 ± 0.26	5.84 ± 5.29	2.50 ± 1.43
IL-7	9.22 ± 4.13	2.13 ± 0.46	5.81 ± 2.38	4.01 ± 0.61
IL-12/IL-23p40	115.21 ± 52.46	186.18 ± 51.77	219.35 ± 64.51	339.62 ± 107.82
IL-15	4.26 ± 0.43	5.03 ± 0.47	5.31 ± 1.05	7.31 ± 1.96
IL-16	219.30 ± 38.11	228.50 ± 36.23	210.22 ± 22.93	414.58 ± 160.38
IL-17A†	2.24 ± 0.83	3.47 ± 1.04	3.92 ± 1.06	6.80 ± 1.27
TNFβ	0.26 ± 0.09	0.23 ± 0.05	0.46 ± 0.17	0.24 ± 0.04
VEGF*	41.06 ± 13.43	15.23 ± 2.00	15.61 ± 3.24	22.08 ± 5.82
Eotaxin	101.07 ± 45.20	126.43 ± 17.00	152.76 ± 35.31	157.62 ± 35.23
Eotaxin3	10.95 ± 4.33	7.46 ± 3.44	4.73 ± 1.38	5.28 ± 1.82
IP10	255.57 ± 112.46	453.98 ± 71.70	663.82 ± 290.99	671.30 ± 146.95
MCP-1‡	113.23 ± 53.56	167.20 ± 18.12	563.49 ± 436.27	221.92 ± 22.80
MCP-4	56.52 ± 29.72	85.69 ± 18.38	86.70 ± 23.59	87.66 ± 19.25
MDC	573.37 ± 140.94	634.85 ± 209.20	599.00 ± 82.56	600.00 ± 143.98
MIP-1α	13.39 ± 2.35	24.88 ± 3.46	26.50 ± 11.28	28.20 ± 6.66
MIP-1β‡	50.59 ± 19.96	89.04 ± 17.07	307.68 ± 249.67	113.73 ± 11.46
TARC	41.75 ± 17.77	50.85 ± 22.40	65.08 ± 20.36	94.28 ± 26.96

$P<0.05$ * interaction between sex and HFrEF, † Effect of sex, ‡ Effects of HFrEF ; IFN, interferon; IL, interleukin; IP-10, interferon-γ–inducible protein 10; MCP, monocyte chemoattractant protein; MDC, C-C motif chemokine 22; MIP, macrophage inflammatory protein; TARC, CCL17/CC motif chemokine ligand 17; TNF, tumor necrosis factor; VEGF, vascular endothelial growth factor.

Table S4: Primers for gene expression used in this study

Gene Symbol	Qiagen Catalog No.	Refseq Accession no.	Reference Position
ACTB	330001 PPH00073G	NM_001101.3	730
ATG7	330001 PPH15687C	NM_006395.2	700
CTSL	330001 PPH00113F	NM_001912.4	375
ESR1	330001 PPH01001A	NM_000125	254
FBXO32 (MAFbx)	330001 PPH19134B	NM_058229.3	1028
HIF-1α	330001 PPH01361B	NM_001530	2418
IGF-1	330001 PPH00167C	NM_000618.4	530
IL-8	330001 PPH00568A	NM_000584.3	326
MAP1LC3B	330001 PPH17765B	NM_022818.4	2091
MSTN	330001 PPH01942E	NM_005259.2	971
MYOD1	330001 PPH00046A	NM_002478.4	391
MYOG	330001 PPH07162C	NM_002479.5	492
NFAT1	330001 PPH00277C	NM_172390.2	2459
NOTCH1	330001 PPH00526C	NM_017617.3	7581
PGC1a	330001 PPH00461F	NM_013261.3	2303
TRIM63 (MuRF1)	330001 PPH15765B	NM_032588.3	669
UBB	330001 PPH17778B	NM_018955.3	1202
VEGFA	330001 PPH 00251C	NM_003376.5	3118

ACTB, Beta actin; ATG7, Autophagy related protein 7; CTSL, Cathepsin-L; ESR1, Estrogen receptor alpha; HIf-1α, Hypoxia-inducible factor 1-alpha; IGF-1, Insulin-like growth factor-1; IL8, Interleukin8; LC3B, Microtubule-associated protein light chain 3; MAFbx, F-box only protein 32; MSTN, Myostatin; MyoD, Myoblast determination protein 1; MYOG, Myogenin; MuRF1, Muscle RING finger 1; NFAT, Nuclear factor of activated T-cells; Notch1, Neurogenic locus notch homolog protein 1; PGC-1α, Peroxisome proliferator-activated receptor-gamma coactivator 1 alpha; UBB, Ubiquitin B; VEGF, Vascular endothelial growth factor

Table S5: Primary and secondary antibodies used in this study.

Antibody	Dilutions	Manufacturer	Cat No:
**AMPKA**	1:1000	Cell Signalling	2532S
**Anti-rabbit IgG HRP linked**	1:1000	Cell signalling	7074S
**Calcineurin**	1:1000	Cell Signalling	2614S
**GAPDH**	1:1000	Cell Signalling	2118S
**MEF2D**	1:1000	Cell Signalling	25651S
**PGC1a**	1:1000	ABCAM	Ab54481
**Phospho-AMPKA**	1:1000	Cell Signalling	2535S
**Phospho-MEF2D**	1:1000	Invitrogen	PA540202
**BA-D5**	14.8:250	DSHB	
**SC-71**	7.6:250	DSHB	
**IgG2b Alexa-Fluor 647nm**	1:500	Invitrogen	A21242
**IgG Alexa-Fluor 488nm**	1:500	Invitrogen	A21121
**UEA I Fluorescein**	1:200	Vector Laboratories	FL-1061
**WGA Rhodamine**	1:1000	Vector Laboratories	RL-1022

AMPK, AMP-activated protein kinase; BA-D5, Myosin heavy chain Type I; GAPDH, Glyceraldehyde 3-phosphate dehydrogenase; HRP, Horseradish peroxidase; IgG, Immunoglobulin G; MEF2D, Myocyte Enhancer Factor 2D; PGC-1α, Peroxisome proliferator-activated receptor-gamma coactivator 1 alpha; SC-71, Myosin heavy chain Type IIA; UEA, Ulex europaeus agglutinin; WGA, Wheat Germ Agglutinin.
